# Application value of CT radiomic nomogram in predicting T790M mutation of lung adenocarcinoma

**DOI:** 10.1186/s12890-023-02609-y

**Published:** 2023-09-11

**Authors:** Xiumei Li, Jianwei Chen, Chengxiu Zhang, Zewen Han, Xiuying zheng, Dairong Cao

**Affiliations:** 1 Department of Radiology, The First Affiliated Hospital of Fujian Medical University, Fuzhou, Fujian 350005 China; 2 Department of Radiology, Fujian Provincial Cancer Hospital, Fuzhou, Fujian 350014 China; 3 Shanghai Key Laboratory of Magnetic Resonance, School of Physics and Electronic Science, East China Normal University, Shanghai200062, China; 4 Department of Radiology, Binhai Campus of the First Affiliated Hospital, National Regional Medical Center, Fujian Medical University, Fuzhou, Fujian 350212 China; 5 Fujian Key Laboratory of Precision Medicine for Cancer, the First Affiliated Hospital, Fujian Medical University, Fuzhou, Fujian 350005 China; 6 Key Laboratory of Radiation Biology of Fujian Higher Education Institutions, the First Affiliated Hospital, Fujian Medical University, Shanghai200062, China

**Keywords:** Lung adenocarcinoma, Radiomics, Computed tomography, T790M

## Abstract

**Background:**

The purpose of this study was to develop a radiomic nomogram to predict T790M mutation of lung adenocarcinoma base on non-enhanced CT lung images.

**Methods:**

This retrospective study reviewed demographic data and lung CT images of 215 lung adenocarcinoma patients with T790M gene test results. 215 patients (including 52 positive) were divided into a training set (n = 150, 36 positive) and an independent test set (n = 65, 16 positive). Multivariate logistic regression was used to select demographic data and CT semantic features to build clinical model. We extracted quantitative features from the volume of interest (VOI) of the lesion, and developed the radiomic model with different feature selection algorithms and classifiers. The models were trained by a 5-fold cross validation strategy on the training set and assessed on the test set. ROC was used to estimate the performance of the clinical model, radiomic model, and merged nomogram.

**Results:**

Three demographic features (gender, smoking, emphysema) and ten radiomic features (Kruskal-Wallis as selection algorithm, LASSO Logistic Regression as classifier) were determined to build the models. The AUC of the clinical model, radiomic model, and nomogram in the test set were 0.742(95%CI, 0.619–0.843), 0.810(95%CI, 0.696–0.907), 0.841(95%CI, 0.743–0.938), respectively. The predictive efficacy of the nomogram was better than the clinical model (*p* = 0.042). The nomogram predicted T790M mutation with cutoff value was 0.69 and the score was above 130.

**Conclusion:**

The nomogram developed in this study is a non-invasive, convenient, and economical method for predicting T790M mutation of lung adenocarcinoma, which has a good prospect for clinical application.

## Introduction

Lung cancer is the leading cause of cancer-related death [1]. Molecular targeted drugs known as epidermal growth factor receptor tyrosine Kinase Inhibitors (EGFR-TKIs) are regarded as standard first-line therapies for advanced EGFR-mutated lung adenocarcinoma contributing to the reduction of lung cancer mortality [2]. First/second-generation TKIs are effective in treating EGFR-mutated lung adenocarcinoma patients due to the longer progression-free survival (PFS) than chemotherapy [3]. Unfortunately, patients inevitably develop drug resistance to TKIs after 1–2 years [4]. The mechanism of drug resistance is secondary gene mutations, such as T790M mutation, MET amplification, and RAS mutation [5]. T790M mutation is the most common acquired drug resistance mutation, accounting for about 50% [6]. T790M mutation has also been detected in a small number of untreated lung adenocarcinoma, known as primary T790M mutation, accounting for about 1% of EGFR-mutated lung adenocarcinoma [7]. Regardless of primary or secondary T790M mutation, third-generation EGFR-TKIs osimertinib is the standard treatment recommended by various authoritative guidelines for patients with T790M mutation. 63% of patients with T790M mutation achieved objective remission, with a median progression-free survival of 9.7 months [8]. Therefore, as long as conditions permit, all patients with locally advanced or metastatic lung adenocarcinoma patients should attempt biopsy to obtain cancer tissue for T790M mutation detection. The biopsy is an invasive procedure and has potential risks such as bleeding, pneumothorax, etc. Although liquid detection is easier and has a lower risk than the biopsy, the sensitivity of liquid detection for T790M mutation is low, and the positive rate varies greatly based on different detection methods [9].

Inspired by omics such as genomics and proteomics, the concept of radiomics was first proposed by Dutch scholar Lambin in 2012 [10]. Radiomics refers to the analysis of massive data to transform the area of interest of the visual image information into high-resolution deep-level characteristic spatial data for quantitative analysis, and then revealing biological information behind the data. Several studies have shown promising results in radiomics in detecting EGFR mutations, ALK mutations, and survival prediction in advanced lung cancer patients [11–13]. As far as we know, there are few reports aimed at predicting T790M mutation in lung adenocarcinoma by radiomics [14, 15]. Therefore, this study is aimed to explore the value of the CT radiomic features-based nomogram in predicting T790M mutations of lung adenocarcinoma.

## Materials and methods

### Studied patient selection

Two institutional review boards from The First Affiliated Hospital of Fujian Medical University and Fujian Provincial Cancer Hospital approved this retrospective study and waived the requirement for written informed consent. Consecutive patients, who were from January 2018 to November 2022, were enrolled in this study from two institutional medical databases. The inclusion criteria were as follows: (1) patients with lung adenocarcinoma were confirmed T790M mutation by gene detection; (2) every patient underwent CT chest examination within 1 week before gene detection; (3) Slice thickness of lung window image ≤ 5 mm. In addition, the exclusion criteria were as follows: (1) The lung window images had image artifacts which can affect the observation; (2) The margin of the tumor was difficult to be delineated. The qualified patients were randomly divided into a training set and a test set in a ratio of 7:3. The demographic data included sex, age, and smoking status.

### T790M mutation analyses

All tumor tissues from surgical resection or biopsy were formalin-fixed and paraffin-embedded. The T790M mutation status was examined by ARMS-PCR. All procedures were performed according to the manufacturer’s protocol.

### CT image acquisition

CT scans of patients from The First Affiliated Hospital of Fujian Medical University were performed on one of the two CT systems (Toshiba: Aquilion CXL 64-slice CT, Aquilion PRIME 80-slice CT). CT scans of patients from Fujian Provincial Cancer Hospital were performed on Philips Brilliance iCT 256-slice Spiral CT. The acquisition and reconstruction parameters were illustrated in Table 1. Interpretation of CT images was done on a lung window (L, − 500; W, 1500) by using a workstation on picture archiving and communication system.

**Table 1:** The acquisition and reconstruction parameters of different CT images

Parameters	The First Affiliated Hospital of Fujian Medical University		Fujian Provincial Cancer Hospital
	Toshiba: Aquilion CXL 64-slice CT	Toshiba: Aquilion PRIME 80-slice CT	Philips Brilliance iCT 256-slice Spiral CT
Tube voltage	120KV		
Tube current	Automatic tube current modulation:100-400mA		
Exposure Time	0.500s	0.500s	0.432s
Detector collimation	64 × 0.625 mm	80 × 0.5 mm	128 × 0.625 mm
Reconstruction thickness	1.0 mm	1.0 mm	2.0 mm
Slice interval	1.0 mm	1.0 mm	1.0 mm
Pitch	0.813	0.813	0.763
Field of view	L	L	L
Matrix	512 × 512	512 × 512	1024 × 1024
Convolution Kernels	FC51	FC51	YA
Voxel size	0.702 mm×0.702 mm×1 mm	0.702 mm×0.702 mm×1 mm	0.684 mm×0.684 mm×2 mm

### CT semantic features analysis

Two radiologists, who have 10 and 15 years of experience in chest imaging diagnosis without knowledge of pathological report information or other information, reviewed lung CT images and determined the following 12 semantic features together: emphysema, pulmonary metastasis, calcification, air bronchogram, cavitation, spiculation, lobulation, halo sign, broncho- vascular convergence, vascular extension, pleural retraction, and pleural effusion. Those semantic features were represented in binary (counted by 0 and 1).

### Radiomic analysis

Manual segmentation of the volume of interest (VOI) in the targeted tumor was performed with 3D slicer software (http://www.slicer. org) by a radiologist with 10 years of experience. The delineation was then reviewed by a radiologist, who has 15 years of experience in chest disease diagnosis and the qualifications to modify the delineation if necessary. All the lesions in both of training and test sets were segmented manually slice-by-slice. The VOI enclosing the lesion was further refined by excluding areas of fat, air, necrosis, and calcification.

We first used BSpline resampled all images in an in-plane resolution of 0.7 mm × 0.7 mm. Then we extracted the features including first-order features, shape features, and texture features. Texture features included gray level co-occurrence matrix (GLCM), gray level size zone matrix (GLSZM), gray level run length matrix (GLRLM), gray level dependence matrix (GLDM), and neighborhood gray-tone difference matrix (NGTDM). Further we applied wave filter (coif 1) and Laplacian of Gaussian filter (LoG, $\sigma$ =1) on the original images to get more features. The filtered image and extracted features were implemented by the PyRadiomics (https://pyradiomics.readthedocs.io/).

50 of 215 cases were selected randomly to estimate the reproducibility of the extracted features. VOIs of these 50 tumors was independently labeled by 10- and 15-years thoracic imaging radiologists. Then the inter-observer correlation coefficients (ICCs) were calculated with a threshold value of 0.75 to select the features with good consistency.

**Fig. 1:**
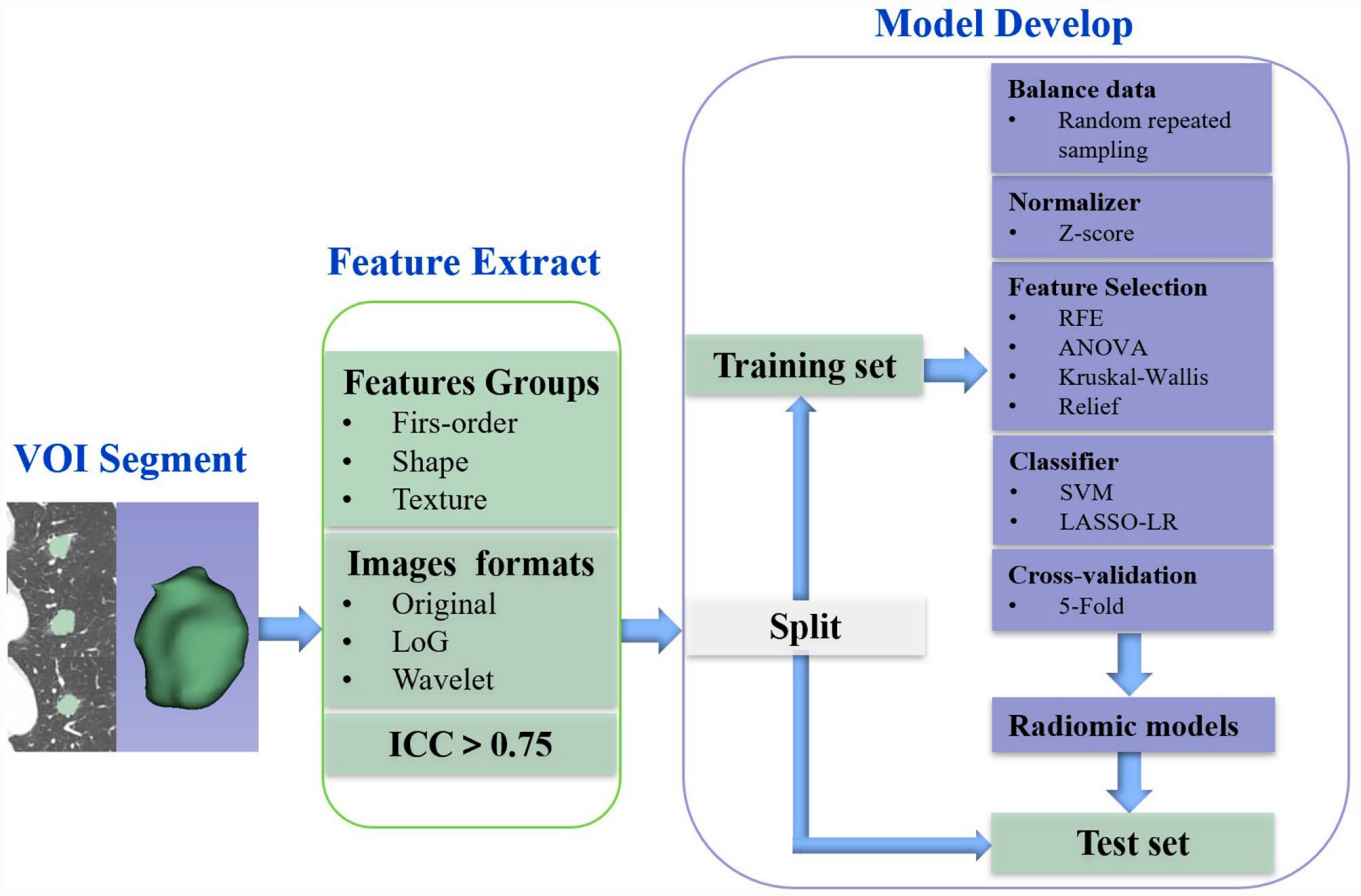
Radiomic analysis workflow

The detailed process of model development was as follows (Fig. 1): for the training data set, we (1) randomly up-sampled the cases to balance data set; (2) normalized each feature to remove the scale variance; (3) removed the redundant features with a cutoff of 0.99 by Pearson Correlation Coefficient (PCC); (4) selected features independently by ANOVA, Kruskal-Wallis, Relief, and Recursive Feature Eliminate; (5) developed the classifier by Support Vector Machine, and LASSO Logistic Regression; (6) applied 5-fold cross validation on the training data set to determine the candidate features and classifier, and (7) built the models using all cases of the training data set. All above were implemented by FAE software (version 0.4.4) [16]. According to the satisfying performance with AUC, the best predictive radiomic model in the test set was selected.

### Establishment of the clinical model and nomogram

The clinical model was built based on the demographics and semantic features by multi-variant analysis. Further we combined the features from clinical model and radscore from radiomic model to construct a nomogram by logistic regression analysis.

### Statistical analysis

We used R statistical software (Version 3.3.3), MedCalc19.0.4, IBM SPSS Statistics 26.0, and Microsoft Excel 2016 for statistical analyses. Continuous variables were expressed as mean ± standard deviation or median (interquartile range) and compared by using the Student’ s t-test or the Mann-Whitney U test. Categorical variables were expressed as numbers and percentages and compared using the Chi-squared or Fisher’s exact test. Two-sided *p*-values < 0.05 was considered statistically significant. Inter-observer correlation coefficients (ICCs) were used to evaluate the agreement of feature extraction. A good agreement was reached when the ICC was greater than 0.75. The area under the curve (AUC) of the receiver operator characteristic curve (ROC), the accuracy (ACC), sensitivity (SEN), specificity (SPE), positive predictive value (PPV), and negative predictive value (NPV) were used to evaluate the model performance. Cut-off value was determined by maximizing Youden index on the training cohort. Decision curve analysis (DCA) was also used to evaluate the clinical usability of the model.

## Results

### Demographic data and CT semantic features analysis

**Fig. 2:**
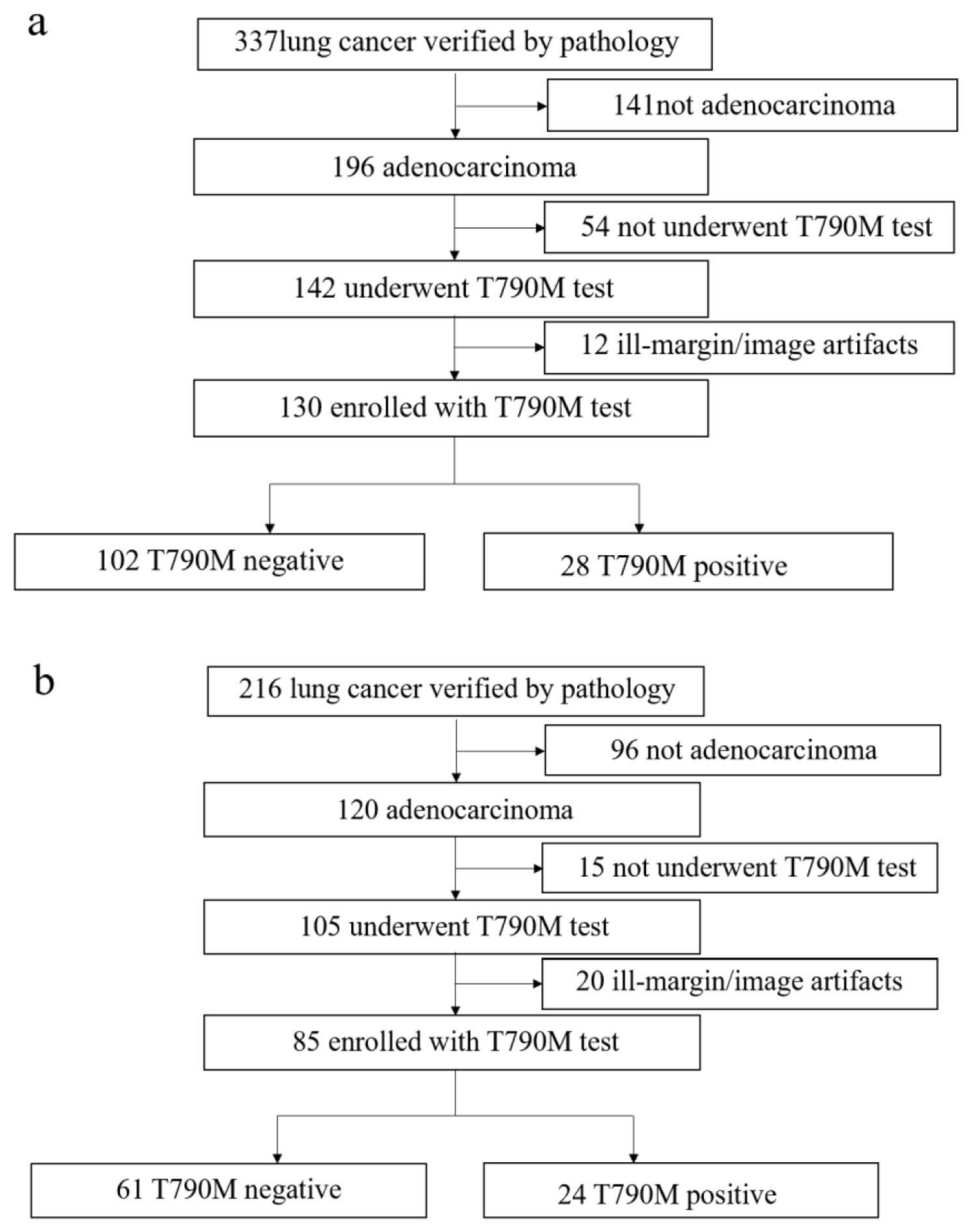
Patient selection flowcharts in (**a**) the First Affiliated Hospital of Fujian Medical University and (**b**) Fujian Provincial Cancer Hospital

A total of 215 patients with lung adenocarcinoma (including 52 patients with T790M mutation) were included in study according to inclusion and exclusion criterions (Fig. 2). Of the 52 patients with T790M mutation, 2 patients were primary mutation and 50 patients were acquired mutation after TKIs therapy. The mean age of the 790 M positive group was 60.27 ± 9.89 years old, and that of the negative group was 63.02 ± 11.56 years old. There was a significant difference in mean age between the positive and negative groups (*p* < 0.001). Pearson’s Chi-square test was used to compare the differences between positive and negative groups in sex, smoking status, location (central/peripheral), and 12 semantic features. The rate of T790M mutation was higher in female than in male (*p* < 0.003), non-smokers than smokers (*p* = 0.009), and non-emphysema than emphysema (*p* = 0.001). Other demographics and semantic features showed no significant difference between the two groups (*p* > 0.05). The results of the correlation analysis in Table 2.

**Table 2:** The results of the correlation analysis between positive and negative groups of T790M mutation

T790M	Cases	Age	Sex		Smoking		Emphysema		Location		Pleural retraction		Vascular extension	
			Male	Female	No	Yes	No	Yes	Central	Peripheral	No	Yes	No	Yes
+	52	60.27 ± 9.89	25	27	43	9	50	2	8	44	34	18	39	13
-	163	63.02 ± 11.56	115	48	103	60	121	42	31	132	113	50	115	48
Z		46.540	8.767		6.880		11.638		0.351		0.283		0.384	
*p*		<0.000	0.003		0.009		0.001		0.554		0.595		0.536	

**Table 2:** **(continued)** The results of the correlation analysis between positive and negative groups of T790M mutation

T790M	Cases	Pulmonary metastasis		Calcification		Air bronchogram		Cavitation		Broncho-vascular convergence		Halo sign		Spiculation		Lobulation		Pleural effusion	
		No	Yes	No	Yes	No	Yes	No	Yes	No	Yes	No	Yes	No	Yes	No	Yes	No	Yes
+	52	35	17	51	1	48	4	51	1	44	8	52	0	14	38	14	38	43	9
-	163	120	43	156	7	154	9	152	11	139	24	156	7	48	115	48	115	145	18
Z		0.781		0.619		0.327		0.742		0.014		2.308		3.338		0.122		1.409	
*p*		0.377		0.431		0.567		0.187		0.907		0.129		0.068		0.726		0.235	

### Clinical model establishment and performance evaluation

**Fig. 3:**
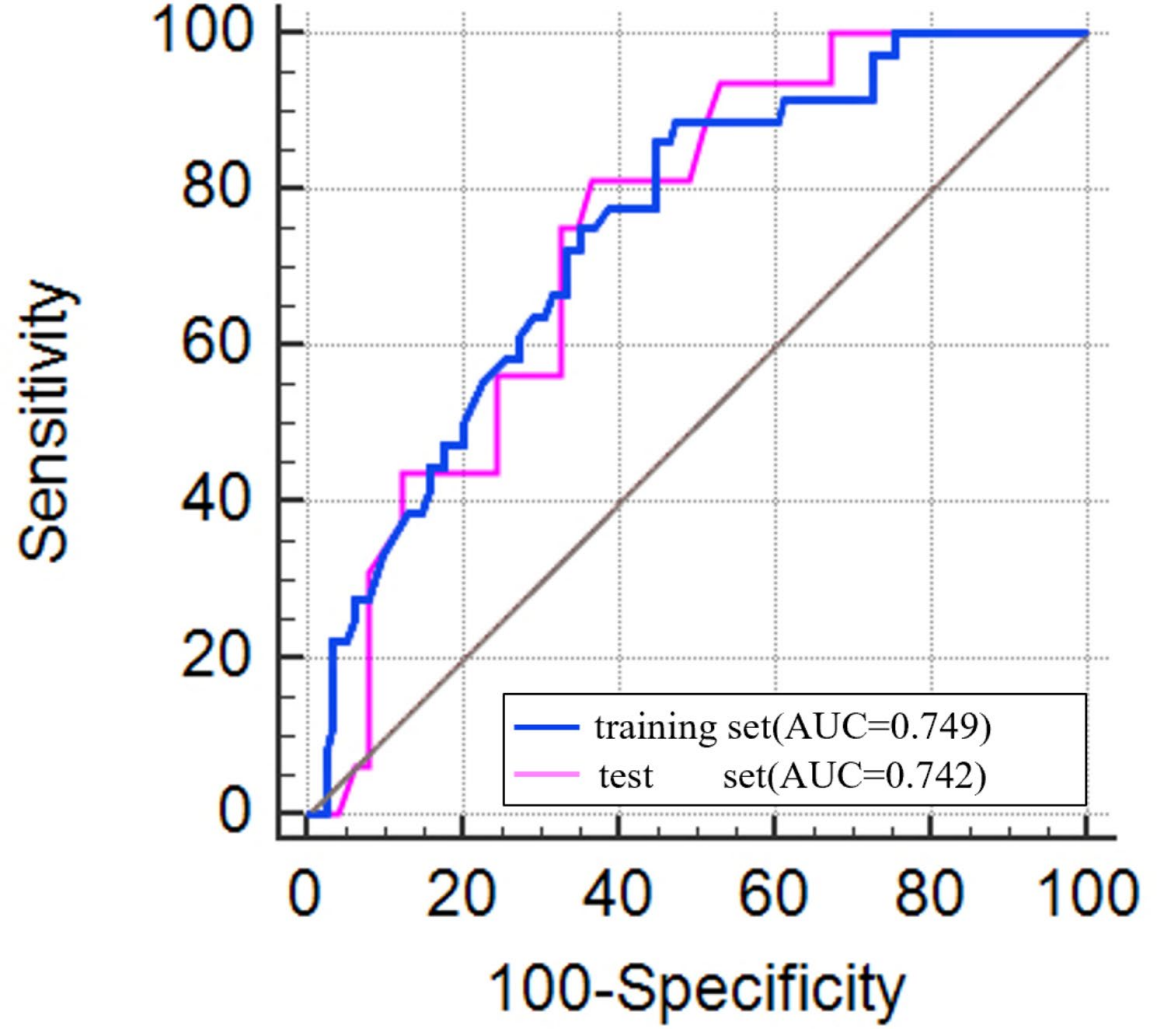
The ROC curves of clinical model in the training and test sets

215 patients were randomly divided into a training set of 150 (36 positive, 114 negative) patients and a test set of 65 (16 positive, 49 negative) patients in a ratio of 7:3. The most distinguishable features containing sex, smoking status, and emphysema status were enrolled to establish a clinical model by multivariate logistic regression. The clinical model score =-0.791 + 0.268× sex (male 0, female 1) -1.163× smoking (no 0, yes 1) -2.086× emphysema (no 0, yes 1). The AUC of the clinical model in the training set was 0.749 (95%CI, 0.672–0.816), and the AUC in the test set was 0.742 (95%CI, 0.619–0.843). The sensitivity, specificity, positive predictive, and negative predictive values were shown in Table 3; Fig. 3.

**Table 3:** The performance of clinical, radiomic, and nomogram models in training and test sets

Model	Set	Case	+/-	AUC	95%CI	Sensitivity	Specificity	Positive predictive value	Negative predictive value	Youden index
Clinical	Training	150	36/114	0.749	0.672–0.816	88.89%	52.63%	37.23%	93.73%	0.415
	Test	65	16/49	0.742	0.619–0.843	81.25%	63.27%	41.91%	91.22%	0.445
Radiomic	Training	150	36/114	0.831	0.756–0.893	86.11%	64.91%	43.77%	93.67%	0.474
	Test	65	16/49	0.810	0.694–0.897	93.75%	67.35%	48.39%	97.06%	0.611
Nomogram	Training	150	36/114	0.892	0.831–0.937	94.44%	74.56%	54.08%	97.76%	0.690
	Test	65	16/49	0.841	0.743–0.938	81.25%	75.51%	52.02%	92.51%	0.568

### Radiomic model establishment and performance evaluation

944 radiomic features were extracted from each VOI of a lesion, including 107 features from the original image, 744 features from the wave filter image, and 93 features from the LoG filter image. A total of 843 features had good consistency between observers with ICCs > 0.75, and were used for model construction. The optimal radiomic model was generated by the following procedures: Z-score was used for features normalization, redundant features with PCC > 0.99 were removed, Kruskal-Wallis was used as the feature selection algorithm, the LASSO logistic regression was used as the classifier to select key features, and the 5-fold cross-validation method was used for model verification. The optimal radiomic model contained 10 radiomic features with non-zero coefficients (Table 4).

**Table 4:** Names and coefficients of non-zero radiomic features

Features name	Coefficient	Mean	Standard deviation
Original_Firstorder_Maximum	0.698	0.907	0.003
Original_Glcm_JointEntropy	0.126	5.653	0.869
Original_Glcm_MaximumProbability	-1.162	1.186	20.131
Wavelet-LHH_Glcm_JointEntropy	-0.197	5.553	0.890
Wavelet-LHH_Gldm_LargeDependenceLowGrayLevelEmphasis	-3.078	0.487	1.903
Wavelet-LHH_Gldm_SmallDependenceHighGrayLevelEmphasis	-0.032	0.770	0.008
Wavelet-LHL_Firstorder_Entropy	-1.160	5.080	1.478
Wavelet-LHL_Glcm_DifferenceVariance	-0.191	0.979	0.129
Wavelet-LHL_Gldm_SmallDependenceHighGrayLevelEmphasis	0.499	0.922	0.009
Wavelet-LHL_Glszm_GrayLevelNonUniformityNormalized	-1.032	2.068	23.469

$$\begin{aligned}{\rm{Radscore}} &  =  - 0.778 + 0.698 \times {\rm{Original}}\_{\rm{Firstorder}}\_{\rm{Maximum}}\\  & \quad  + 0.126 \times {\rm{Original}}\_{\rm{Glcm}}\_{\rm{JointEntropy}}\\  & \quad  - 1.162 \times {\rm{Original}}\_{\rm{Glcm}}\_{\rm{MaximumProbability}}\\  & \quad  - 0.197 \times {\text{Wavelet-LHH}}\_{\rm{Glcm}}\_{\rm{JointEntropy}}\\  & \quad  - 3.078 \times {\text{Wavelet-LHH}}\_{\rm{Gldm}}\_{\rm{LargeDependenceLowGrayLevelEmphasis}}\\  & \quad  - 0.032 \times {\text{Wavelet-LHH}}\_{\rm{Gldm}}\_{\rm{SmallDependenceHighGrayLevelEmphasis}}\\  & \quad  - 1.160 \times {\text{Wavelet-LHL}}\_{\rm{Firstorder}}\_{\rm{Entropy}}\\  & \quad  - 0.191 \times {\text{Wavelet-LHL}}\_{\rm{Gcm}}\_{\rm{DifferenceVariance}}\\  & \quad  + 0.499 \times {\text{Wavelet-LHL}}\_{\rm{Gldm}}\_{\rm{SmallDependenceHighGrayLevelEmphasis}}\\  & \quad  - 1.032 \times {\text{Wavelet-LHL}}\_{\rm{Glszm}}\_{\rm{GrayLevelNonUniformityNormalized}}\\ \end{aligned}$$

**Fig. 4:**
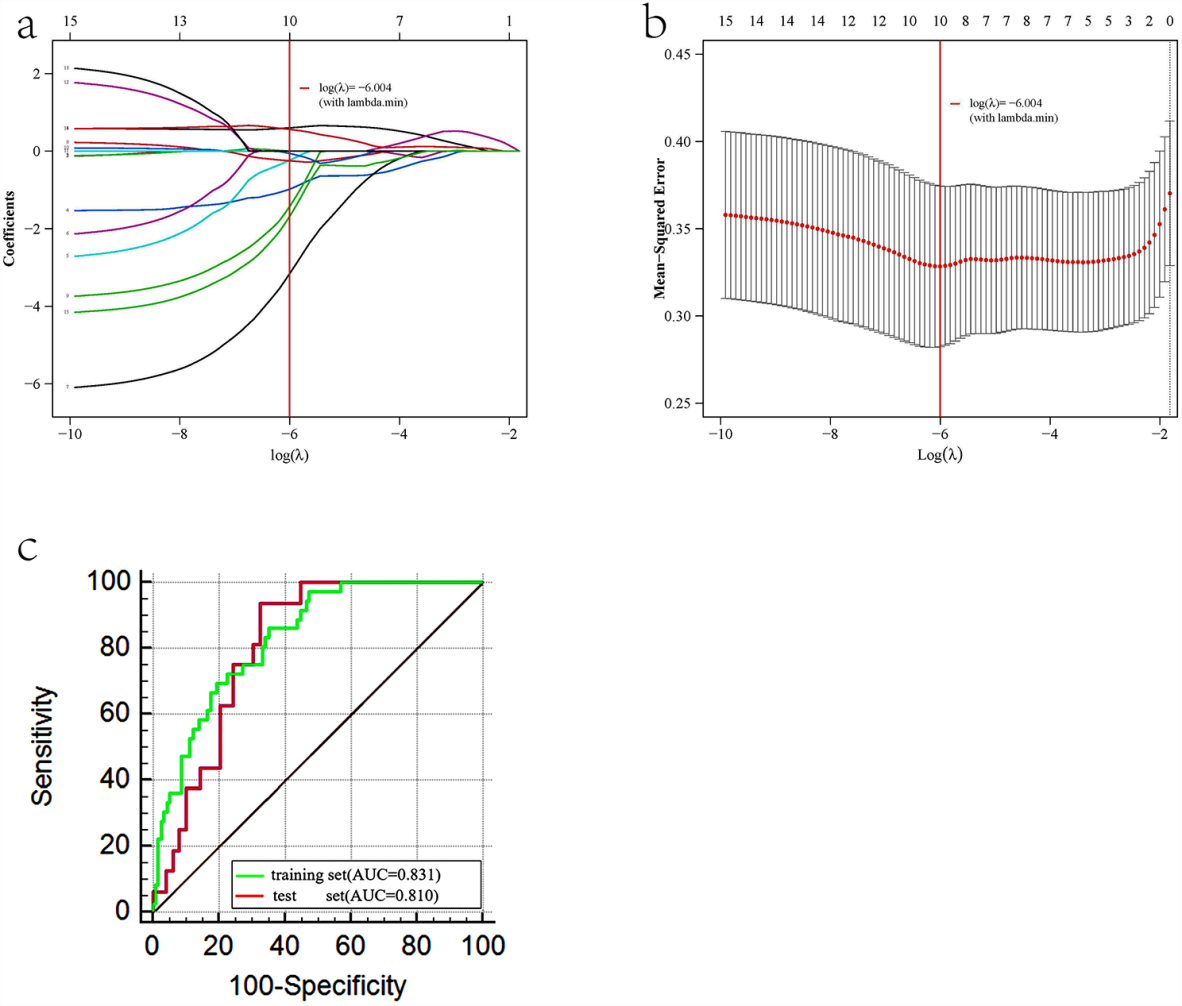
(**a**, **b**) LASSO dimensionality reduction curve of radiomic features. (**c**) The ROC curves of the radiomic model in the training and test sets

The AUC of the radiomic model in the training and test sets were 0.831 (95%CI, 0.756–0.893) and 0.810 (95%CI, 0.696–0.907), respectively, as shown in Table 3; Fig. 4.

### Nomogram establishment and performance evaluation

**Fig. 5:**
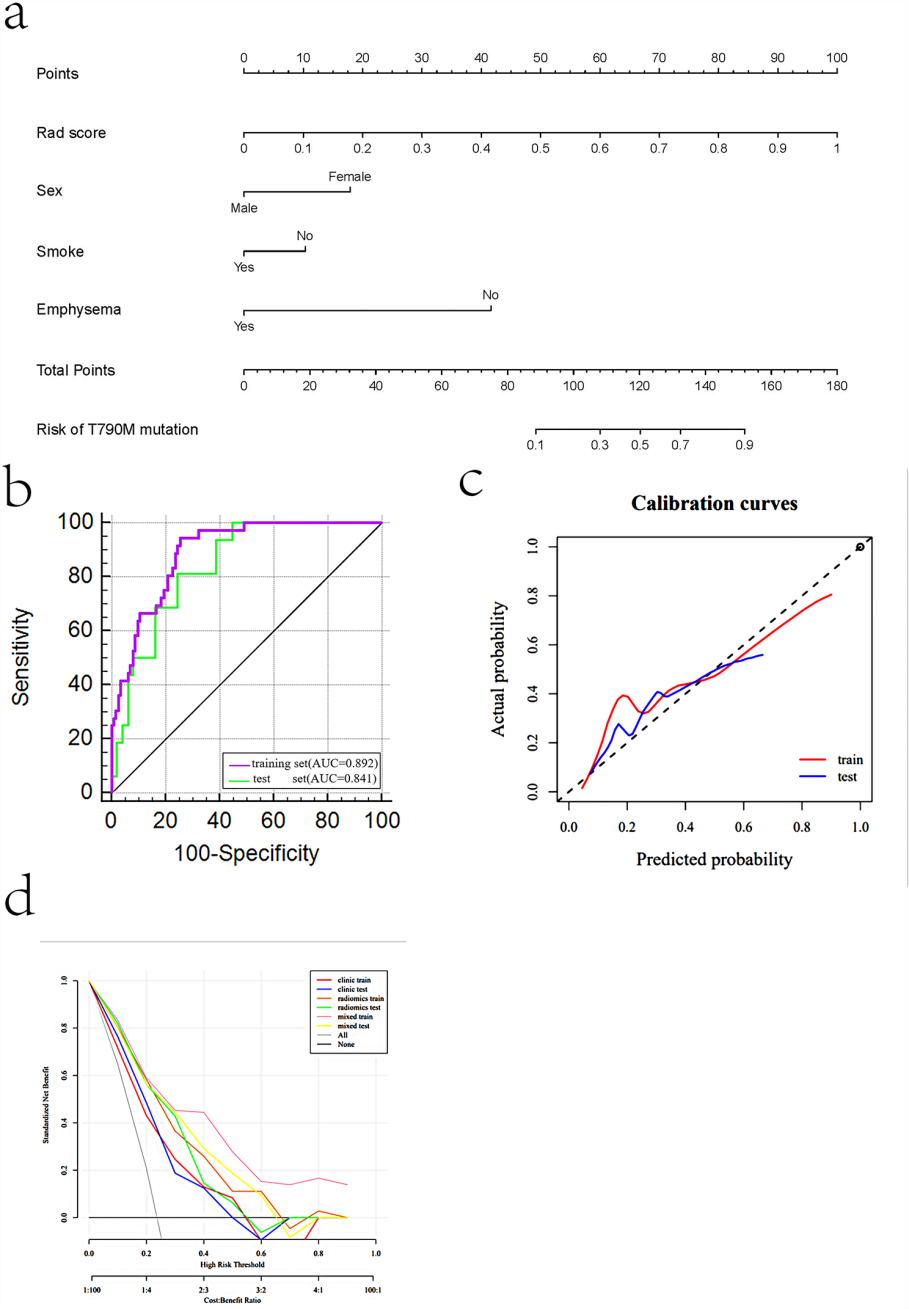
(**a**) The nomogram for predicting T790M mutation was built in the training dataset with the radiomic features, sex, smoking, and emphysema. T790M mutation rate of 69% corresponded to a total score of 130. (**b**) The ROC curves of the nomogram in the training and test sets. (**c**) Nomogram calibration shows good calibration performance in both the training set and the test set. (**d**) The decision curve analysis showed that the nomogram performed best than the clinical and radiomic models

Statistically significant clinical features, such as sex, smoking, emphysema and radiomic radscore were included in the multivariate logistic regression analysis to establish a nomogram for predicting T790M mutation. Nomogram score = -4.738 + 1.240× sex (male 0, female 1) -0.715× smoking (no 0, yes 1) -2.886× emphysema (no 0, yes 1) + 6.935×radscore. The AUC of the nomogram in the training and test sets were 0.892 (95%CI, 0.831–0.937) and 0.841 (95%CI, 0.743–0.938), respectively, as shown in Table 3. Nomogram calibration shows good calibration performance in both the training set and the test set, the decision curve analysis showed that the nomogram performed is the best one among 3 models, as shown in Fig. 5. According to the maximum Youden index of the ROC curve of the training set, the cut-off value of the line graph was determined to be 0.69, corresponding to a total score of about 130 points. When the prediction probability value > 0.69, the probability of T790M mutation increases. The Hosmer-Lemeshow test showed no statistical significance in the diagnostic efficiency of the nomogram in both the training set (*p* = 0.850) and the test set (*p* = 0.206).

**Fig. 6:**
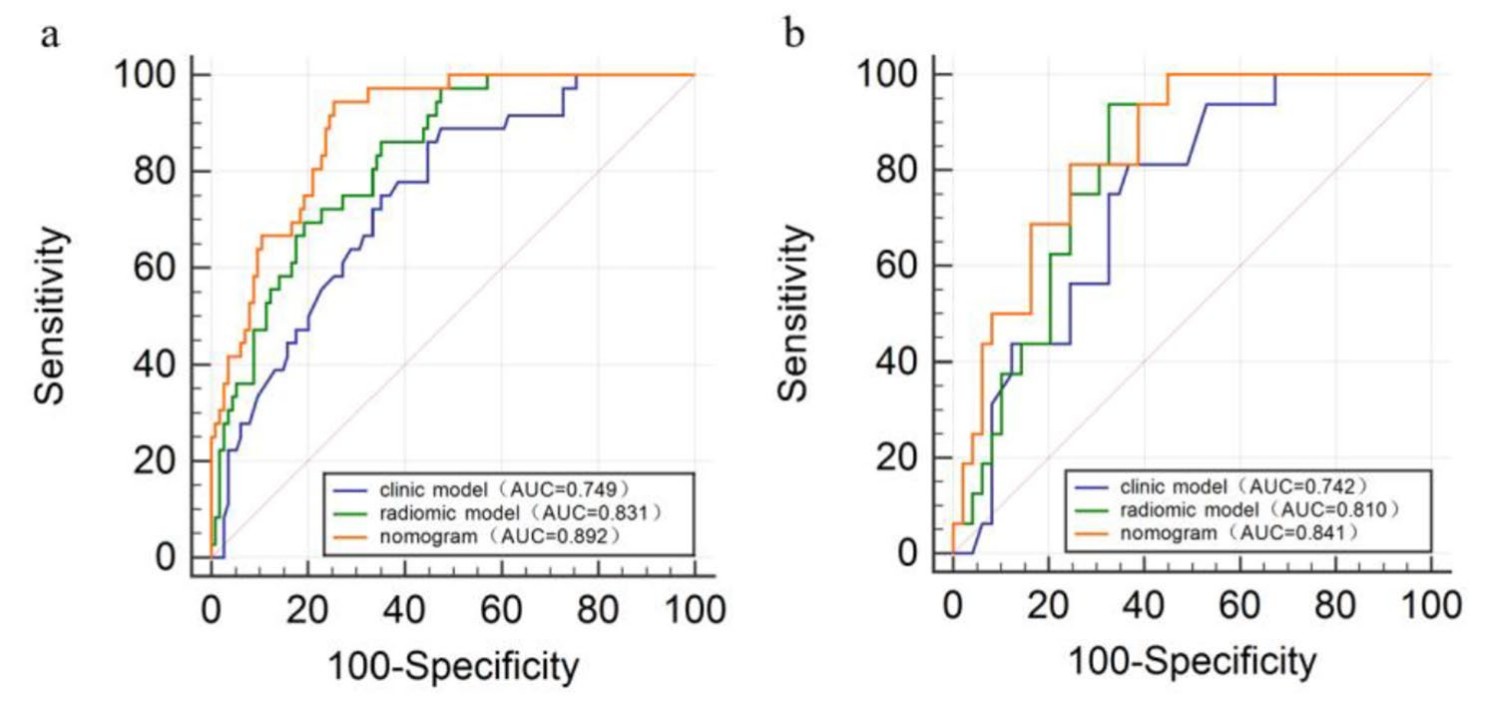
(**a**) The ROC curves of the clinical model, radiomic model, and nomogram in the training set. The AUC of the nomogram was statistically different from that of the clinical model (Z = 3.181, *p* = 0.002) and the radiomic model (Z = 2.350, *p* = 0.019) (**b**) The ROC curves of the clinical model, radiomic model, and nomogram in the test set. The AUC of the nomogram was statistically different from that of the clinical model (Z = 2.032, *p* = 0.042), while there was no statistically difference between the nomogram and the radiomic model (Z = 0.627, *p* = 0.531)

In the training set, the AUC of the nomogram was statistically different from that of the clinical model (Z = 3.181, *p* = 0.002) and the radiomic model (Z = 2.350, *p* = 0.019), as shown in Fig. 6a. In the test set, the AUC of the nomogram was statistically different from that of the clinical model (Z = 2.032, *p* = 0.042), while there was no statistically difference between the nomogram and the radiomic model (Z = 0.627, *p* = 0.531), as shown in Fig. 6b.

## Discussion

This study found that T790M mutation is more likely to occur in female, non-smoking, non-emphysema lung adenocarcinoma patients, and these clinical features are as same as EGFR mutation, which was reported in the literature [17–19]. This can be interpreted as follows: (1) T790M mutation belongs to the range of EGFR mutation. (2) Recent studies have shown that T790M positive cells are also generated from T790M negative single cells in the original EGFR gene through genetic evolution [20]. This study showed that CT semantic features had no predictive effect on T790M mutation. Donghui Hou’s team [7] showed that compared with acquired T790M mutation, primary T790M mutation was more likely to occur in patients with multiple lesions, ground glass density, air bronchogram, and cavitation in lesions. We hold the opinion that osimertinib is the optimal treatment for both primary and secondary acquired T790M mutation patients with advanced lung adenocarcinoma, so there is no necessary to distinguish primary or secondary acquired T790M mutation. Moreover, there were only two cases of primary T790M mutation in this study, so there was no comparison of the signs between primary and secondary in positive T790M patients. In this study, the predictive efficacy AUC of the clinical model in the training set and the test set were 0.749 and 0.742, respectively. However, since EGFR mutation is more likely to occur in female, non-smoking, non-emphysema lung adenocarcinoma patients, we only predict the EGFR mutation not T790M mutation in clinical practice.

In this study, it was shown that the radiomic features based on non-enhanced CT images were helpful to identify the status of T790M mutations, and the AUC of its predictive efficacy in the training set and the test set were 0.831 and 0.810, respectively, which was better than the results of previous studies [19, 21, 22]. Previous studies showed a significant correlation between radiological phenotypes and T790M mutations (*p* = 0.07) [22]. Some studies have proposed that although enhanced images can obtain more stable features, their ability to reflect tumor texture heterogeneity is not as good as that of non-enhanced images, which reduce the interference of contrast agents on tumor heterogeneity, and there is no clear relationship between the stability of features and the accuracy of the model [23]. Lan He’s study showed non-enhanced CT-based radiomic signature demonstrated better discrimination and classification capability than contrast-enhanced CT in both primary and validation cohort [23]. In this study, patients do not need to inject CT contrast agents, which is more conducive to the application in patients with liver and kidney function impairment.

We combined the features of clinical model and radiomic model based on the CT non-enhanced images to construct the nomogram to predict the T790M mutation in patients with advanced lung adenocarcinoma. The predictive efficacy of the nomogram was 0.892 in the training set, which was better than the clinical (0.749) and radiomic (0.831) models. The predictive efficacy of the nomogram was 0.841 in the test set, which was better than the clinical (0.742) model. In terms of obtaining the status of the T790M gene, the nomogram is more non-invasive, low-cost, and convenient than liquid biopsy and tissue biopsy detection. Radiomic profiling and liquid testing are both non-invasive methods to obtain genetic status, but they have limitations when used alone. The sensitivity of liquid testing is not ideal, and radiomics is difficult to interpret in the absence of biological correlation. A study [22] has shown that combining clinical characteristics, radiomic features, and liquid biopsy detection results can better predict the mutation status of T790M, which will be the direction for our further research.

Several studies have reported that the detection of low frequency T790M-positive clones in pre-treatment clinical specimen [20, 24]. The same result appeared to be in our study, of 52 T790M positive patients, 50 patients were acquired mutation after TKIs therapy accounting for 99.96%, 2 were primary mutations accounting for 0.04%. Although therapy might affect radiomic features, it does not affect the clinical practice of the nomogram developed in this study due to T790M acquired mutations after treatment account for the majority.

We also noted the limitations of this study: (1) Since this is a retrospective study, the information about histological growth patterns, TNM stages, and overall grading might improve the performance of the nomogram, but it was imperfect. (2) There is no external data to verify the generalization of the nomogram, and more comprehensive multi-center data verification is needed. (3) Some studies have shown that the therapeutic benefit of T790M-targeted EGFR-TKIs is proportional to the proportion of cells carrying T790M mutation [25]. This study did not further explore the relationship between radiomic features and the abundance of T790M mutations. (4) The VOIs of lesions were segmented by manual segmentation, which is time-consuming. The above deficiencies should be the content and direction of our further research.

## Conclusion

The nomogram developed in this study based on radiomic features from non-enhanced CT images is a non-invasive, convenient, and economical method for predicting the T790M mutation of lung adenocarcinoma. It can detect gene status during treatment and has a good clinical application prospect in helping clinicians to establish faster and more reliable treatment decisions.

## Data Availability

The datasets used and/or analyzed during the current study are available from the corresponding author on reasonable request.

## Declarations

### Statement

All methods in this study were carried out in accordance with relevant guidelines and regulation under the ‘Ethics approval and consent to participate’.
